# Have we achieved adequate recommendations for target volume definitions in anal cancer? A PET imaging based patterns of failure analysis in the context of established contouring guidelines

**DOI:** 10.1186/s12885-019-5970-0

**Published:** 2019-07-29

**Authors:** Hendrik Dapper, Kilian Schiller, Stefan Münch, Jan C. Peeken, Kai Borm, Wolfgang Weber, Stephanie E. Combs

**Affiliations:** 1 Department of Radiation Oncology, Klinikum rechts der Isar, TU München, Ismaninger Str. 22, 81675 Munich, Germany; 2 Institute for innovative Radiotherapy (iRT), Helmholtz Zentrum München, Ingolstädter Landstr. 1, Neuherberg, Germany; 3 Department of Nuclear Medicine, Klinikum rechts der Isar, TU München, Ismaninger Str. 22, 81675 Munich, Germany; 4 Deutsches Konsortium für Translationale Krebsforschung (DKTK), Partner Site Munich, Munich, Germany

**Keywords:** Anal cancer, PET-CT, PET-MRI, Radiation therapy, Contouring guidelines, Target volume, Inguinal contouring recommendations

## Abstract

**Background:**

There are different contouring guidelines for the clinical target volume (CTV) in anal cancer (AC) which vary concerning recommendations for radiation margins in different anatomical regions, especially on inguinal site. PET imaging has become more important in primary staging of AC as a very sensitive method to detect lymph node (LN) metastases. Using PET imaging, we evaluated patterns of LN spread, and examined the differences of the respective contouring guidelines on the basis of our results.

**Methods:**

We carried out a retrospective study of thirty-seven AC patients treated with chemoradiation (CRT) who underwent FDG-PET imaging for primary staging in our department between 2011 and 2018. Patients showing PET positive LN were included in this analysis. Using a color code, LN metastases of all patients were delineated on a template with “standard anatomy” and were divided indicating whether their location was in- or out-field of the standard CTV as recommended by the Radiation Therapy Oncology Group (RTOG), the Australasian Gastrointestinal Trials Group (AGITG) or the British National Guidance (BNG). Furthermore, a detailed analysis of the location of LN of the inguinal region was performed.

**Results:**

Twenty-two out of thirty-seven AC patients with pre-treatment PET imaging had PET positive LN metastases, accumulating to a total of 154 LN. The most commonly affected anatomical region was inguinal (49 LN, 32%). All para-rectal, external/internal iliac, and pre-sacral LN were covered by the recommended CTVs of the three different guidelines. Of forty-nine involved inguinal LN, fourteen (29%), seven (14%) and five (10%) were situated outside of the recommended CTVs by RTOG, AGITG and BNG. Inguinal LN could be located up to 5.7 cm inferiorly to the femoral saphenous junction and 2.8 cm medial or laterally to the big femoral vessels.

**Conclusion:**

Pelvis-related, various recommendations are largely consistent, and all LN are covered by the recommended CTVs. LN “misses” appear generally cranially (common iliac or para-aortic) or caudally (inguinal) to the recommended CTVs. The established guidelines differ significantly, particular regarding the inguinal region. Based on our results, we presented our suggestions for CTV definition of the inguinal region. LN involvement of a larger number of patients should be investigated to enable final recommendations.

## Background

Definitive radiotherapy with concomitant chemotherapy (CRT) is the standard treatment for locoregional squamous-cell carcinoma of AC patients. This procedure has been established by large prospective trials [1, 2]. Primary tumors bigger than 5 cm and involved locoregional LN have been identified as the most important prognostic factors for locoregional recurrence, distant metastases and overall survival [3]. AC patients present with positive or uncertain LN status in about 20 and 10%, respectively. Not surprisingly, 5-year OS in node negative patients has been shown to be superiorly compared to nodal positive patients (63% vs. 37%) [4]. In the largest prospective trials (UKCCCR, ACT II) overall LN involvement was 32%. About 10–25% of all AC patients present with synchronous and another 5–25% with metachronous inguinal LN metastases [5–7].

Especially due to uncertainties regarding LN involvement, the role of ^18^F-fluorodeoxyglucose (FDG) positron emission tomography computed tomography (PET-CT) and PET magnetic resonance imaging (PET-MRI) became more important for primary staging in recent years. In locoregional advanced situations PET-CT is recommended for staging and planning of definitive CRT [8]. A meta-analysis of twelve studies dealing with pretreatment PET-CT could prove PET-CT to be more sensitive (99%) compared to CT-scan alone (60%), leading to a notable amount of upstaging (15%) or downstaging (15%). Nodal staging and TNM-stage changed in 28 and 41% [9].

There are different established contouring guidelines for AC referring to intensity modulated radiotherapy (IMRT) [8, 10–12]. Although these guidelines provide solid evidence and reproducibility in day-to-day radiation therapy, there are still differences in the definition of elective radiation volumes in some anatomical regions. Especially for inguinal nodes, it is known that there is still a lack of evidence regarding field margins [11].

In an analysis of prostate cancer patients treated with primary radiation, it has been shown that more than one third of PSMA-PET-CT positive LN would have been outside of the CTV recommended by RTOG consensus [13]. Similarly, in AC, the use of initial PET-CT after MRI significantly altered the radiation volume [14]. In the present study, we analysed patterns of spread of involved LN at primary diagnosis of AC based on FDG-PET imaging (PET-CT or PET-MRI) and correlated the results with established guidelines for delineation of target volumes in AC. We sought to determine if LN detected by PET are predominantly included in the CTV of guidelines and if there are critical subsites for marginal misses. Finally, we proposed our suggestions for CTV definition of the inguinal region.

## Methods

Between 2011 and 2018, thirty-seven AC patients who were treated with CRT in our institution underwent FDG-PET imaging, either CT or MRI based, for primary staging of cancer of the anal canal or anal margin. Compliance with ethical standards was met. Inclusion criteria for our study were:

- confirmed squamous cell anal carcinoma by biopsy
- at least one positive LN metastasis on FDG-PET-CT/MRI

Exclusion criteria were:

- metastatic disease (except of common iliac or para-aortic LN metastases)
- previous surgical intervention or radiation therapy in the pelvis

Due to these criteria, twenty-two out of thirty-seven patients were selected for further analysis and are described in this study. All patients underwent MRI imaging for T-stage definition and detailed primary tumor localization. Staging was performed according to the “TNM Classification of Malignant Tumors – Eighth Edition” [15]. Two patients had common iliac LN metastases and three patients had common iliac LN and para-aortic LN metastases. Contrast-enhanced FDG-PET imaging was either performed as PET-CT (*n* = 18; Biograph mCT scanner, Siemens Medical Solutions, Germany) or an integrated whole-body PET-MRI system (*n* = 5, Siemens Biograph mMR, Siemens Medical Solutions, Germany) after intravenous injection of FDG. In one patient, a PET-CT and a PET-MRI were performed. Median activity of F − 18-FDG was 311 MBq (range: 236–655 MBq) and the median interval between injection and start of PET acquisition (“uptake time”) accounted for 81 min (range: 60–108 min). The examined field extended from the scull base to the proximal femoral. In one patient with PET-MRI, the detection area included only the abdomen and the pelvis. All patients received oral contrast enhancement. In eleven and four patients additional rectal contrast agent was administered. Twelve of the eighteen patients with PET-CT scan had a diagnostic CT scan of 3 mm slice thickness. In seven cases, low dose CT attenuation correction was needed. The used MRI sequences amounted at least axial/sagittal T2 TSE, axial DWI, axial T1 TSE −/+ and sagittal T1. MRI reconstruction was in 3 mm slice thickness. We carried out quantitative evaluation of attenuation-corrected image data by standardized uptake value (SUV calculation.

PET-CT/MRI reading and interpretation were performed by two experienced nuclear medicine physicians/radiologists. Basically, pelvic LN from 1.0 cm and inguinal LN from 1.5 cm in diameter were considered suspect. However, for the definition of PET-positivity of LN, the combination of different factors such as SUV values, morphology and size of the LN as well as other prognostic factors, such as the tumor stage, were considered.

**Fig. 1:**
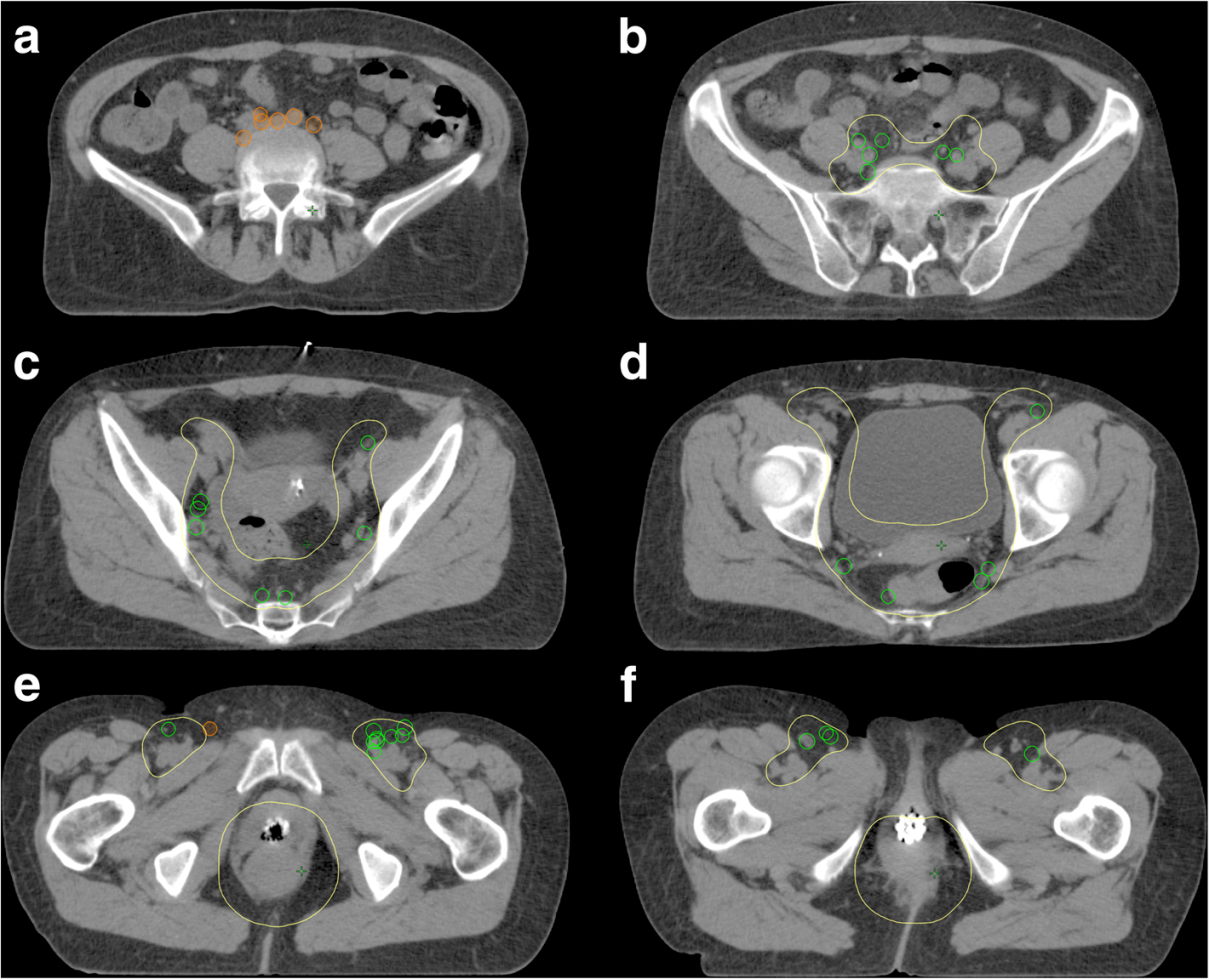
Elective CTV (yellow) as recommended by the RTOG in different CT-slices defined on a standard anal cancer case. Green circles = infield LN. Orange circles = outfield LN.1a: ultimately above the common iliac joint at the height of L5; 1b: first cranial slide at the level of the common iliac joint; 1c: inclusion of external iliac, internal iliac LN and the pre-sacral space above the urinary bladder; 1d: transition of the inguinal and external iliac nodes (lower level of internal obturator artery) with inclusion of the mesorectum, pre-sacral space and the internal iliac LN. Advanced margins (1 cm) into the urinary bladder; 1e: height of the symphysis. Coverage of the inguinal nodes and the anal canal with 2 cm safety margin; 1f: caudal border of the inguinal LN (2 cm below the saphenous/femoral junction) and the primary tumor on primary site

To obtain an overview of the anatomical distribution of all PET-positive LN of all different patients at the same time, we developed a method to transfer all involved LN on a single CT scan. This was carried out analogously to Schiller et al., who have performed a similar evaluation in prostate cancer [13]. As a first measure, we selected a planning CT scan (3 mm slices thickness) for radiation therapy of a certain AC patient with “standard anatomy” (female, body mass index: 21.7) as a template. Secondly, the three different CTVs of the current international recommendations were contoured on this CT. The first CTV was defined regarding to the recommendations of RTOG (see Fig. 1) [8]. The second CTV was delineated analogously to the contouring guidelines of the AGITG and the third to those of the BNG [11, 12]. The RTOG and AGITG guidelines for IMRT of AC could be identified via PubMed search using “Contouring guidelines anal cancer”. The BNG is an evidence based consensus for IMRT of AC and currently standard of care within the UK. It is used within the PLATO trial. As the next step, all PET-positive LN of the twenty-two patients were delineated on the one chosen CT scan (template) by an experienced radiation oncologist. To transfer the LN to the template as accurately as possible, the anatomical conditions of each positive LN in the original PET imaging of all twenty-two patients were considered (relations to e.g. vessels or musculoskeletal structures). LN locations were defined as inguinal, external and internal iliac (including obturator nodes), pre-sacral, para-rectal, common iliac and para-aortic, and were recorded in a table (Table 1). The LN were contoured by standard starting from the centre of the LN consistently on three axial CT slices (longitudinal extension: 9 mm) by using a brush with 9 mm diameter to represent each LN at 9 × 9 mm. Afterwards, the radiation oncologist evaluated whether these LN were covered by the three CTVs of the different contouring guidelines. This was done individually for each of the three CTVs. The definition of “miss” arose from the fact that the majority (> 50%) of the volume of the LN was not covered by the CTV. Using a color code, the LN metastases were divided indicating whether their location was in- (green) or out-field (orange) of the standard CTV. The process of LN transfer to the template and the decision as to whether a LN was predominantly included within a particular CTV, was reviewed by at least one other experienced radiation oncologist.

**Table 1:** PET-positive LN in anal cancer patients and LN outside the CTV using different contouring guidelines

Location/ Patient	1	2	3	4	5	6	7	8	9	10	11	12	13	14	15	16	17	18	19	20	21	22	Σ
T-stage	T4	T3	T2	T2	T2	T2	T3	T3	T3	T2	T4	T3	T3	T3	T4	T2	T4	T4	T2	T2	T1	T2	
*Para-aortic*		**6**		**6**													**1**						**13**
RTOG		6		6													1						13
AGITG		6		6													1						13
BNG		6		6													1						13
*Common iliac*		**13**		**1**							**1**						**1**		**1**				**17**
RTOG		12		0							0						1		0				13
AGITG		12		0							0						1		0				13
BNG		12		0							0						1		0				13
*External iliac*		**2**		**11**			**1**			**2**			**1**		**2**	**1**	**3**	**3**					**26**
RTOG		0		0			0			0			0		0	0	0	0					0
AGITG		0		0			0			0			0		0	0	0	0					0
BNG		0		0			0			0			0		0	0	0	0					0
*Internal iliac*		**1**		**5**	**1**		**1**		**1**			**1**		**1**	**1**		**4**						**16**
RTOG		0		0	0		0		0			0		0	0		0						0
AGITG		0		0	0		0		0			0		0	0		0						0
BNG		0		0	0		0		0			0		0	0		0						0
*Pre-sacral*		**3**		**1**		**1**			**1**		**2**				**1**		**2**				**2**	**1**	**14**
RTOG		0		0		0			0		0				0		0				0	0	0
AGITG		0		0		0			0		0				0		0				0	0	0
BNG		0		0		0			0		0				0		0				0	0	0
*Peri-rectal*	**1**	**2**				**1**		**2**	**1**	**1**	**1**	**2**	**1**			**2**				**2**	**1**	**2**	**19**
RTOG	0	0				0		0	0	0	0	0	0			0				0	0	0	0
AGITG	0	0				0		0	0	0	0	0	0			0				0	0	0	0
BNG	0	0				0		0	0	0	0	0	0			0				0	0	0	0
*Inguinal*	**2**	**5**	**1**	**14**						**1**	**1**		**1**		**6**		**2**	**16**					**49**
RTOG	0	3	0	6						0	0		1		0		0	4					14
AGITG	0	1	0	3						0	0		1		0		0	2					7
BNG	0	0	0	3						0	0		1		0		0	1					5
**Total LN**	**3**	**32**	**1**	**38**	**1**	**2**	**2**	**2**	**3**	**4**	**5**	**3**	**3**	**1**	**10**	**3**	**13**	**19**	**1**	**2**	**4**	**3**	**154**

Each column corresponds to one patient. LN outside the recommended CTV of RTOG = Radiation Oncology Group, AGITG = Australasian Gastrointestinal Trials Group and BNG = British National Guidance. Bold = total number of positive LN.

Due to larger differences in the three contouring guidelines with respect to the inguinal region, a detailed evaluation of the location of the inguinal LN was performed. The individual LN were assigned to the exact LN region described in a standard anatomy atlas [16]. Further, the shortest radial distance of the LN (measured from the centre of the LN) to the big vessels (femoral vein and artery, great saphenous vein) and the longitudinal distance to the inferior CTV margins of the three recommendations were measured.

Statistical analysis was conducted using ‘IBM SPSS statistics’ software, version 23.0 (IBM, Armonk, USA). A Chi-Square test was applied to analyse differences regarding T-stage and the distribution of LN outside or inside the CTV of RTOG.

## Results

### Patients’ characteristics and patterns of LN involvement

**Fig. 2:**
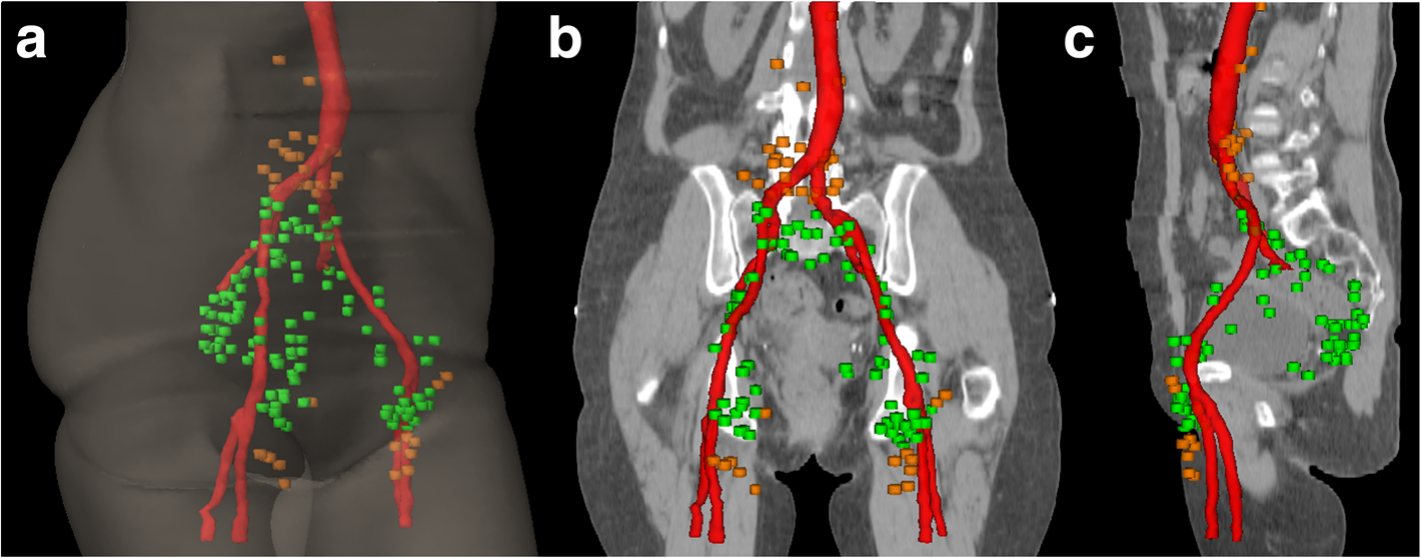
PET-positive LN at primary diagnosis of twenty-two anal cancer patients. Green = LN which were properly covered by the elective CTV of the RTOG. Orange = LN which were outside the elective CTV of the RTOG

Twenty-two out of thirty-seven patients (59%) had PET-positive LN metastases. About two third (68%) of these twenty-two patients were female. Median and mean age at diagnosis was 62 years. T2 (8), N1a (12) stage IIIC (9) and G2 (12) were the most common tumor characteristics. In twenty patients the tumor was predominantly locolized in the area of the anal canal, whereas only two patients had a primary cancer of the anal verge. However, the tumor reached the anal margin in another three patients. A total of 154 FDG-PET positive LN were found (Table 1). The mean and median number of involved LN per patient was seven and three (range: 1–34). The most commonly affected anatomical region was inguinal (49 LN, 32%). Furthermore, we found nineteen para-rectal, fourteen pre-sacral, sixteen internal iliac, twenty-six external iliac, seventeen common iliac and thirteen para-aortic LN. An overview of the exact position of all LN and the information whether those were in- or outfield of the CTV recommended by RTOG is illustrated in Fig. 2 *and* Table 1.

### LN outside the CTV

Forty (26%), thirty-three (21%) and thirty-one (20%) of all LN were outside the CTVs of RTOG, AGITG and BNG. All were found in five patients (23%). These patients had stage T2 (1), T3 (2) and T4 (2) tumors. Four of them had extensive locoregional disease with more than twelve LN in nearly every anatomical subsite of the pelvis and inguinal. The LN which were not covered by standard CTV were located para-aortic (13), para iliac common (13) and inguinal (RTOG: 14; AGITG: 7; BNG: 5). These LN, except of three inguinal, were caudally (11) or cranially (26) of the CTV. No misses were found inside the pelvis (peri-rectal, pre-sacral, external and internal iliac).

### Inguinal LN

We found forty-nine PET-positive inguinal LN in ten of twenty-two patients (45%) (Table 2). These were distributed as follows: eighteen profound (deep), thirteen inferior, thirteen superomedial and six superolateral superficial inguinal LN. Fourteen (29%), seven (14%) and five (10%) inguinal LN were not properly covered by the CTV of RTOG, AGITG and BNG. Two superolateral and one superomedial misses occurred regardless of which of the three CTV definitions was used. However, there were differences in the lower part of the inguinal region. Ten LN (20%) were located more than 2 cm inferiorly to the saphenous/femoral junction (RTOG), whereas only four LN (8%) were below the level of the lesser tuberosity (AGITG) and just two (4%) below the lesser trochanter (BNG). The deepest LN was less than 5.5 cm below the saphenous/femoral junction. The mean radial distance from the LN to the vessels amounted to 1.3 cm (range: 0.4–2.8 cm). Thirty-one LN (63%) kept more than 1 cm distance. Especially the superomedial and superolateral superficial LN might have had more than 1.5 cm distance to the femoral vessels (15 LN ≥1.5 cm). In those patients with LN below the saphenous/femoral junction (inferior LN group), the LN all were in between 10 mm to the great saphenous vein, whereas the profound LN were usually very close to the femoral vessels. With their outer shape, nine LN reached more than 5 mm to the skin of the medial thigh.

**Table 2:** Detailed location of PET-positive inguinal LN at primary diagnosis of anal cancer

Pat. No	LN No	LN station		Cranio-caudal distance in cm				Vessels radial distance in cm		
			LN Diameter	Saphenous junction (RTOG)	Anal verge	Lesser tuberosity (AGITG)	Lesser trochanter (BNG)	Fem.	Saph.	Skin
1	1	inferior	1.2	−1	+ 2.1	+ 2.4	+ 3	1.2	0.6	0.8
	2	inferior	1.4	-1	+ 2.1	+ 2.4	+ 3	1.4	0.7	1.1
2	3	superomedial	1.7	0	+ 0.9	+ 1.5	+ 2.4	2.1	–	0.9
	4	superomedial	1.6	−3	−0.5	0	+ 1.0	2.4	0.8	1.2
	5	inferior	1.5	−4	−1.5	− 0.5	0	1.4	0.9	1.2
	6	inferior	1.3	−3	−0.5	+ 0.5	+ 1.0	2.1	1.0	0.7
	7	inferior	1.0	−1	+ 1.0	+ 1.5	+4.0	0.8	0.9	1.6
3	8	superomedial	1.0	−0.3	3.3	3.3	3.9	1.1	0.5	0.9
4	9	inferior	1.2	−5.5	−2.5	−2.5	−0,5	1.8	0.7	1.6
	10	inferior	2.6	−3	0	0	+ 2	2.4	1.0	2.1
	11	inferior	1.0	-3	0	0	+ 2	1.7	0.7	2.5
	12	inferior	1.0	−2.5	+ 1.0	+ 0.5	+ 2.5	0.8	0.6	2.9
	13	profound	2.6	0	+4	+ 3	+4.5	1.7	1.3	1.2
	14	superolateral	2.1	+ 1.5	+ 5	+4	+ 6	2.6	–	0.8
	15	superolateral	0.8	+4.5	+ 7	+ 6.0	+ 7.5	2.8	–	0.6
	16	inferior	1.0	−2	+ 3	+ 1.5	+4.0	0.7	1.1	2.9
	17	superomedial	2.3	−1.2	+ 3.6	+ 4.1	+ 5.7	1.4	1.3	1.1
	18	profound	0.6	+ 3.3	+ 6.3	+ 7.2	+ 8.9	0.4	–	3.9
	19	profound	1.0	+4.5	+ 7.5	+ 7.8	+ 9.3	0.8	–	4.5
	20	profound	0.6	+ 1.3	+4.3	+4.8	+ 6.7	0.4	–	2.8
	21	profound	1.0	+ 2.9	+ 5.4	+ 5.4	+ 7.3	0.6	–	3.0
	22	profound	0.9	+ 3.2	+ 6.4	+ 7.3	+ 8.9	0.7	–	2.4
5	23	superomedial	0.9	−1.4	+ 5.5	+ 3.4	+ 5.4	0.7	0.7	2.7
6	24	superomedial	1.1	+ 1,4	+ 7.5	+ 5.1	+ 6.6	0.7	0.7	0.9
7	25	superomedial	1.4	+ 2.5	+ 7.4	+ 6.3	+ 6.4	2.5	–	2.8
8	26	superomedial	1.3	−1.6	+ 3,6	+ 3,6	+ 3,6	1.4	0.6	2.9
	27	profound	1.9	−1.8	+ 3.9	+ 3.9	+ 3.7	1.3	1.0	1.6
	28	profound	2.1	0	+4.3	+4.5	+4.4	1.1	1.5	3.1
	29	profound	1.2	0	+ 5.2	+ 5.2	+ 5.3	1.9	1.5	2.4
	30	superolateral	1.4	+ 2.3	+ 5.5	+ 5.4	+ 5.4	1.7	–	2.1
	31	profound	1.0	+ 2.0	+ 5.3	+ 5.3	+ 5.4	0.6	–	5.5
9	32	superomedial	1.3	+ 2.1	+ 5.1	+ 5.6	+ 7.0	0.7	–	0.7
	33	profound	1.6	+ 1.6	+ 5.6	+ 5.0	+ 6.4	0.9	–	0.9
10	34	inferior	1.4	−5.7	−2.6	−0.7	−0.2	2.8	0.8	1.3
	35	inferior	1.3	−5.6	−2.6	−0.6	0	2.3	0.7	1.5
	36	inferior	1.5	−2.8	−0.7	+ 1.3	3.2	1.3	0.8	1.3
	37	inferior	1.2	−2.8	−0.7	+ 1.4	+ 3.3	1.4	0.8	1.5
	38	superomedial	0.9	−1.9	+ 1.3	+ 3.4	+4.3	1.7	0.4	1.3
	39	superolateral	1.2	− 0.6	+ 3.1	+ 5.1	+ 6	1.3	–	0.7
	40	profound	1.0	+ 1.5	+ 3.9	+ 5.8	+ 6.9	0.7	–	0.8
	41	profound	1.0	+ 2.5	+4.9	+ 6.8	+ 7.9	0.6	–	1.5
	42	inferior	0.8	−1.9	+ 1.4	+ 3.4	+4.3	0.7	0.4	1.0
	43	profound	1.2	−1.6	+ 2.1	+4.1	+ 5.1	0.8	1.0	3.0
	44	superomedial	1.0	+ 1.5	+ 3.9	+ 5.9	+ 7.0	1.5	–	1.4
	45	superomedial	0.9	+ 2.4	+4.9	+ 6.9	+ 7.8	1.4	–	1.1
	46	profound	0.8	+ 2.4	+4.9	+ 6.9	+ 7.9	1.5	–	0.8
	47	profound	0.7	+ 2.3	+4.8	+ 6.8	+ 7.8	1.3	–	0.3
	48	superolateral	0.7	+ 3.4	+ 5.3	+ 7.3	+ 8.3	0.9	–	0.6
	49	superolateral	0.8	+ 3.9	5.9	+ 7.8	+ 8.9	1.1	–	0.6

LN = Lymph node, Pat. = Patient, No = number, cm = centimetre, Saph. = great saphenous vein, Fem. = femoral vessels. RTOG = Radiation Oncology Group, AGITG = Australasian Gastrointestinal Trials Group, BNG = British National Guidance. + = cranial distance, − = caudal distance. Red = LN mainly outside elective CTV.

Dependent on the T-stage, LN showed a significantly different distribution of being outside or inside of the CTV of RTOG.

## Discussion

PET imaging is a very sensitive method for detecting LN metastases in primary staging of AC [9]. We evaluated the patterns of spread of LN in patients with primary diagnosis of AC. Forty (26%), thirty-three (21%) and thirty-one (20%) of 154 out of these LN were located outside the CTV recommendations of RTOG, AGITG and BNG. Concerning the inguinal region, differences between the three guidelines in terms of LN misses were thus shown. Especially regarding the inguinal region and the ischiorectal fossa, blatant differences between these CTVs exist. The main differences are demonstrated in Table 3. In the following, we discuss our findings of LN spread in context with the margins of the three guidelines.

**Table 3:** Summary of elective CTV recommendations of different contouring guidelines for IMRT in critical regions of anal cancer

	CTV delineation recommendations		
	Cranial (internal & external iliac nodes/mesorectal)	Caudal (inguinal)	Ischiorectal fossa
RTOG 2009 [8]	**Mesorectal** - Rectosigmoid junction or 2 cm superior to superior extent of gross disease (rectum/perirectal nodes) **Internal & external iliac nodes** - The most cephalad aspect of CTV: bifurcation of common iliac vessels into external/internal iliacs (approximate boney landmark: sacral promontory)	- Always elective coverage of inguinal and external iliac region - inferior: **2 cm caudal to the saphenous/femoral junction**. - “The inguinal/femoral region should be contoured as a compartment with any identified nodes (especially in the lateral inguinal region) included.”	- If no tumor extension into ischiorectal fossa: CTV just a few millimetres beyond the levator muscles - Advanced anal, extending through the mesorectum or the levators: “~ 1–2 cm margin up to bone wherever the cancer extends beyond the usual compartments.”
BNG 2016 [12]	**Internal & external iliac nodes** - Cranial internal, external iliac and pre-sacral space: “bifurcation of the common iliac artery into the external and internal iliac arteries (usually corresponds to the L5/S1 interspace level)” **Mesorectal** - If no mesorectal nodes: The lower 50 mm of the mesorectum. - If involved mesorectal nodes: The level of the recto-sigmoid junction	- Should be added as a compartment - Superficial and deep inguinal nodes of the femoral triangle and visible benign LN or lymphoceles outside these boundaries. - Borders: lateral: medial edge of sartorius or ilio-psoas, medial: spermatic cord in men. Posterior: pectineus, adductor longus and iliopsoas. Anterior: 5 mm from skin. Inferior: **lesser trochanter**.	No direct recommendations for the ischiorectal fossa. CTV gross tumor of locally advanced tumors: - CTV_A = GTV + 15 mm
AGITG 2011 [11]	**Internal & external iliac nodes** “Cranial: bifurcation of the common iliac artery into the external and internal iliac arteries (usually corresponds to the L5/S1 interspace level)” “The sacral promontory, defined at the L5/S1 interspace” **Mesorectal** “Cranial: the level of the recto-sigmoid junction; best identified where the rectum runs anteriorly to join the sigmoid colon (Atlas 4b).”	- Inclusion of superficial and deep inguinal LN of the femoral triangle and any visible LN or lymphoceles. Borders: inferior: “there is no consensus”, so compromise: **lower edge of the ischial tuberosities**. Posterior: muscles, anterior: minimum 20-mm margin on the inguinal vessels, including any visible LN or lymphoceles, lateral: medial edge of sartorius or iliopsoas, medial: a 10- to 20-mm margin around the femoral vessels. The medial third to half of the pectineus or adductor longus muscle serves as an approximate border.	- Cranial: levator ani, gluteus maximus, and obturator internus, caudal: suggestion: level of the anal verge. Lateral: ischial tuberosity, obturator internus, and gluteus maximus muscles. Anterior: fusion of anal sphincters. Inferiorly: 10 to 20-mm anterior to the sphincter muscles. Posterior: a transverse plane joining the anterior edge of the medial walls of the gluteus maximus muscle.

### Pelvis

All three guidelines consequently demand the inclusion of the mesorectum, the obturator nodes, the pre-sacral space and the external and internal iliac nodes. After defining the different CTVs, none of the positive LN (0/71) was located outside of either the elective volumes.

*Ischiorectal fossa*: The RTOG consensus group “agreed that, unless there is radiographic evidence of extension into the ischiorectal fossa, extension of CTV does not need to go more than a few millimetres beyond the levator muscles”, whereas the AGITG recommend an inclusion of the whole ischiorectal fossa. In our analysis, we could not identify any LN laterally beyond the levator muscles inside the ischiorectal fossa. This indicates, as recommended by the RTOG and BNG, that the ischiorectal fossa does not need to be included in the elective target volume if the levator muscle is not involved.

### Cranial border

In all three guidelines, the cranial border of the elective CTV in anal cancer is the bifurcation of the common iliac artery into the external and internal iliac arteries. Additionally, the pre-sacral space should be included up to this height, which is important, since we have some history in marginal misses with IMRT in this subside [17, 18]. After the evaluation of twenty-two patients with PET-positive LN, a reasonable number of common iliac (17) and para-aortal (13) LN could be identified. However, para-aortic LN occurred in only three patients who had multiple (> 12) and also common iliac LN metastases which were already visible in the native CT or MRI scan. In those cases, a PET-CT scan is highly recommended before an individual curative intended CRT is initiated to exclude distant metastases and to define a proper “individually adapted” target volume [8]. Two patients had common iliac nodes but no para-aortic LN. Summarized, the level of common iliac junction would be a sufficient cranial margin as long as PET imaging would be performed in patients with locally advanced disease in initial MRI or CT. Patients with involved para-aortic LN in the absence of distant metastases might be treated with CRT as definitive therapy [19].

### Inguinal

The biggest differences between the three contouring guidelines exist regarding the inguinal region (Table 3). We had five to fourteen inguinal misses depending on which margins were used. Hence it is not surprizing that, up to now, there is no evidence for consistent and reproducible recommendations of an elective target volume in this region. This is also mentioned by the AGITG [11]. From the radio-oncological point of view, it is inconsistent that the ano-inguinal lymphatic drainage is not described and included into the elective CTV, although recent immunofluorescence studies have presented reasonable anatomical definitions for this drainage [20, 21]. The anatomy in the inguinal region is very complex due to large differences between the individuals. Therefore, it is all the more important to correlate the target volume with basic anatomy. The clinical classification of the different inguinal LN groups can be divided by a cross with an oblique horizontal axis. The vertical axis corresponds to the femoral vessels and the oblique horizontal axis runs along the lower edge of the inguinal ligament. The cross divides the LN into four inguinal groups, each with different positional relationship to the big vessels [22].

*Dorsal and dorsolateral*: all three contouring guidelines enclose the space between the inguinal/femoral vessels and the muscles (pectineus, adductor longus, iliopsoas and the medial edge of sartorius or ilio-psoas). In our analysis, interestingly, none of the fourty-nine LN was located dorsal or lateral to the vascular tracts in the space to the thigh muscles (Fig. 2b, c). This small space might be excluded from the CTV.

**Fig. 3:**
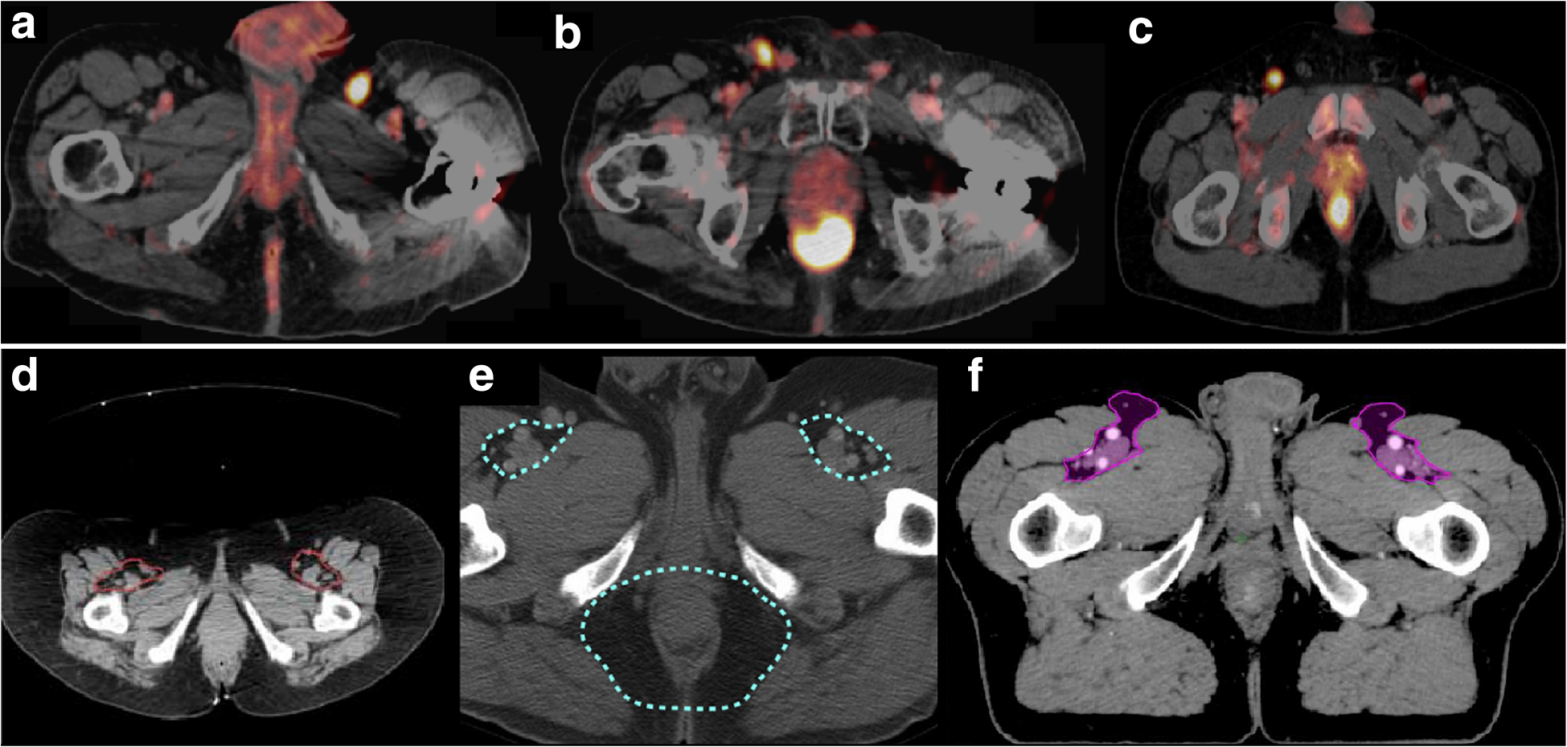
PET-positive superomedial superficial inguinal LN (**a**–**c**) in anal cancer patients (SUV_max_ /SUV_mean:_ 3a: 13.3/8.4; 3b: 6.0/3.6; 3c: 5.9/3.4). Those were not properly covered by the elective CTV recommendations of RTOG (d) and AGITG (**e**) but completely included in the CTV of BNG (**f**)

*Medial, lateral, ventral*: the RTOG recommends contouring of the inguinal region “as a compartment with any identified nodes”. This formulation is understandable due to interindividual anatomical differences but inconclusive as some nodes may have a considerable distance to the vessels or are not even seen on CT scan. The guidelines of the AGITG and BNG give more detailed field borders. The AGITG recommend anteriorly a minimum of 20 mm margin on the inguinal vessels, lateral the medial edge of sartorius or iliopsoas and medial a 10–20 mm margin around the femoral vessels, even if this is not implemented consistently in the example (Fig. 3e). The BNG recommend laterally the same, anteriorly up to 5 mm from the skin and medial any visible LN or lymphocele or the spermatic cord in men. We could identify quite a high number of superomedial (13) and superolateral (6) superficial inguinal LN with a distance of up to 2.8 cm to the big vessels. Many of these LN were laterally or medially just partially covered by the CTV and five LN were total misses. Figure 3a–c gives an example of three superomedial superficial LN which are critical and which are most probably not properly covered in an elective CTV by the recommendations of the RTOG (Fig. 3d) and AGITG (Fig. 3e). The BNG recommends a medial extension to the spermatic cord in men, which would be sufficient to cover the involved superomedial nodes. The conclusion from our patient sample is that a medio-ventral margin of 3 cm along the genital vessels would include all superomedial LN. Just the RTOG guidelines consequently include the superficial superolateral LN group (named as lateral LN). The medial edge of m. sartorius as lateral margin (AGITG, BNG) would lead to a reasonable number of failures. These could be avoided by the inclusion of 0.5–1 cm of the ventral space of the medial part of the sartorius (3 cm from the femoral vessels). Finally, the lateral borders of the RTOG and the medial borders of the BNG seem to be a reasonable solution. Although the CTV might reach ventrally the femoral skin in many patients, the skin up to 5 mm (BNG) does not seem to be a useful recommendation, as we could not identify any involved LN near to the skin in slightly obese patients. Further, a large CTV would cause an inappropriately high toxicity. We could define radial margins from the vessels which would have covered all LN satisfactorily by using cm-margins (2 cm from the femoral vessels, 1 cm from the great saphenous vein).

*Inferior*: there is insufficient evidence for the inferior inguinal border, as mentioned by the AGITG [11]. The fact that the ano-inguinal lymphatic drainage is located on the medial thigh and can fall very deep (about 3 cm) below the level of the anal verge, was recently shown with the help of the immune fluorescence method [20, 21]. Therefore, the three guidelines have different recommendations for inferior inguinal margins. The RTOG defines the caudal margin “2 cm caudal to the saphenous/femoral junction”, the BNG determines the “lesser trochanter” and the AGITG identifies “the lower edge of the ischial tuberosities” as most inferior extension of the CTV. In the analysed collective, ten misses (20% of all nodes) occurred inferiorly to the CTV of the RTOG. Only four LN were located below the lower edge of the ischial tuberosity and two LN below the lower edge of the lesser trochanter. The patient with very caudal inguinal misses had a T4 tumor which had already infiltrated the left labia, and should be seen as a special individual case. However, also patients with a T2 tumor and no infiltration to the anal border had inguinal LN below 2 cm inferiorly the saphenous/femoral junction.

Due to anatomical diversity and the ano-inguinal lymphatic drainage, we would relate the inferior inguinal border in patients with no involvement of the anal margin to the level of the anal verge. If the tumor affects anal margin or extensive disease or multiple suspected LN (≥5 LN), the inferior border should be 2 cm below the anal verge. Furthermore, the ano-inguinal lymphatic drainage should be added [20, 21].

### Patterns of recurrence

To find best possible recommendations for CTV definition for locoregional advanced AC, patterns of recurrence should also be considered. We could not identify any meta-analysis dealing with patterns of recurrence in IMRT/VMAT treated AC patients. Most recurrence studies stem from the 3D era [23, 24]. The biggest and most detailed IMRT-analysis of patterns of recurrence is from Tomasoa et al. and included 106 patients. About one fifth of the collective developed a recurrence within a time interval of two to seventy-one months (median 15 months). The vast majority of recurrence was local in the anus or rectum (14/106, 13%). Such relapses seem to occur due to insufficient dose prescription in aggressive AC and cannot be attributed to inappropriate CTV definition. Only two LN recurrences occurred in the pelvis (pelvic side wall, probably obturator, and pre-sacral) and were most likely marginal misses. Tomasoa et al. did not find any recurrence above the level of S3. This could be explained by the fact that PET was performed in most cases. Despite inguinal radiation, the inguinal side was the only LN region with a reasonable number of recurrences (4 patients, 4%) [25]. Unfortunately, it was not reported whether those LN were marginal misses or clearly outfield. These results correspond with our findings that the CTV definition in the inguinal region should be optimized.

### Limitations

We had some limitations in this study. There were no strict and thus reproducible criteria by which LN were finally classified as involved. A certain degree of uncertainty (false positive/negative) is, however, inevitable since a final assessment always has to take various factors into consideration. Another difficulty was the correct transfer of positive LN of twenty-two patients on one patients’ planning CT scan. Using a standard size for involved LN on one patients’ data-set distorted the situation of some individual cases. However, we were able to relativize this problem by measuring distances in millimetre to various relevant structures. Special care has to be taken when interpreting the inguinal misses in patients with extensive locoregional situations which are defined as metastatic disease (M1, LYM). We included these patients as they were treated in curative intention with a standard protocol of CRT. These cases are not representative and therefore basically not useful to derive a meaningful elective CTV definition for all patients. In principle, guidelines serve as an orientation for a reasonable standardized target volume in order to cover potential micrometastases and to save regions with very low risk of tumor invasion to reduce toxicity. Of course, these prescriptions are abandoned in real clinical scenarios when macrometastases appear in the imaging (e. g. para-aortic LN would be included). However, these locoregional advanced cases provide fundamental reference for possible anatomical patterns of inguinal involvement as it can be assumed that some of these PET positive LN were already affected but not visible or morphologically suspicious at an earlier point in time with clinically lower stage. Furthermore, the judgment whether a LN would ultimately be inside or outside of a particular CTV is difficult. It would be presumptuous to assume that up to 26 mm large LN would not have been included in the CTV by a radiation oncologist, although these LN were just outside the recommendations by established guidelines. In addition, the phrases such as “the inguinal/femoral region should be contoured as a compartment with any identified nodes (especially in the lateral inguinal region) included” used by the RTOG leave a great deal of scope for inter-individual CTV definitions. Accordingly, the real “misses” cannot be correctly recorded with any method. Finally, these formulations and also the large inaccuracies regarding the described CTV and the contoured CTV (for all three guidelines) as well as large inter-individual differences in the contouring of the CTV (RTOG) showed that evidence regarding the contouring of the inguinal region is urgently required. Of course, there is the possibility of false positive LN. However, the number is very difficult to ascertain, because studies on this topic and thus the final proof by histological assurance are missing.

The strength of our study was the provision of the assignment of affected LN to relevant anatomical structures with millimetre-precise distances in the original patient. Based on these results we were able to make specific distance-based guidance for the contouring of the inguinal region.

## Conclusion

In this study, we demonstrated patterns of LN involvement based on PET imaging. In the pelvis, various recommendations are largely consistent and all LN were covered by the recommended CTVs. LN “misses” appear generally cranially (common iliac or para-aortic) or caudally (inguinal) to the recommended CTVs. The established guidelines differ significantly, particular regarding the inguinal region. Based on our results, for CTV-definition in the inguinal region, we generally would suggest a 2 cm radial margin from the large femoral vessels and 1 cm from the saphenous/femoral junction. To cover the superomedial and superolateral superficial LN, a medial and lateral CTV-margin of 3 cm along the lower inguinal ligament seems to be meaningful. The caudal border should be at the level of the anal margin. Patterns of LN involvement of a large number of patients should be investigated to enable final guidelines.

## Data Availability

The present data is summarized in this paper (Methods). The complete dataset can be retrieved from the authors upon formal request of interested readers.

## Abbreviations

AC: Anal Cancer
AGITG: Australasian Gastrointestinal Trials Group
BNG: British National Guidance
CRT: Chemoradiation
CT: Computed Tomography
CTV: Clinical Target Volume
IMRT: Intensity Modulated Radiotherapy
LN: Lymph node
MRI: Magnetic Resonance Imaging
PET: Positron Emission Tomography
RTOG: Radiation Therapy Oncology Group
SUV: Standardized uptake value
